# Schizophrenia Detection and Classification: A Systematic Review of the Last Decade

**DOI:** 10.3390/diagnostics14232698

**Published:** 2024-11-29

**Authors:** Arghyasree Saha, Seungmin Park, Zong Woo Geem, Pawan Kumar Singh

**Affiliations:** 1 Department of Information Technology, Jadavpur University, Jadavpur University Second Campus, Plot No. 8, Salt Lake Bypass, LB Block, Sector III, Salt Lake City, Kolkata-700106, West Bengal, India; arghyasree07@gmail.com (A.S.); pksingh.it@jadavpuruniversity.in (P.K.S.); 2 Department of Software, Dongseo University, Busan 47011, Republic of Korea; 3 College of IT Convergence, Gachon University, Seongnam 13120, Republic of Korea

**Keywords:** schizophrenia detection, machine learning, EEG signals, MRI scans, deep learning, artificial intelligence, systematic review

## Abstract

Background/Objectives: Artificial Intelligence (AI) in healthcare employs advanced algorithms to analyze complex and large-scale datasets, mimicking aspects of human cognition. By automating decision-making processes based on predefined thresholds, AI enhances the accuracy and reliability of healthcare data analysis, reducing the need for human intervention. Schizophrenia (SZ), a chronic mental health disorder affecting millions globally, is characterized by symptoms such as auditory hallucinations, paranoia, and disruptions in thought, behavior, and perception. The SZ symptoms can significantly impair daily functioning, underscoring the need for advanced diagnostic tools. Methods: This systematic review has been conducted following the PRISMA (Preferred Reporting Items for Systematic Reviews and Meta-Analyses) 2020 guidelines and examines peer-reviewed studies from the last decade (2015–2024) on AI applications in SZ detection as well as classification. The review protocol has been registered in the International Prospective Register of Systematic Reviews (PROSPERO) under registration number: CRD42024612364. Research has been sourced from multiple databases and screened using predefined inclusion criteria. The review evaluates the use of both Machine Learning (ML) and Deep Learning (DL) methods across multiple modalities, including Electroencephalography (EEG), Structural Magnetic Resonance Imaging (sMRI), and Functional Magnetic Resonance Imaging (fMRI). The key aspects reviewed include datasets, preprocessing techniques, and AI models. Results: The review identifies significant advancements in AI methods for SZ diagnosis, particularly in the efficacy of ML and DL models for feature extraction, classification, and multi-modal data integration. It highlights state-of-the-art AI techniques and synthesizes insights into their potential to improve diagnostic outcomes. Additionally, the analysis underscores common challenges, including dataset limitations, variability in preprocessing approaches, and the need for more interpretable models. Conclusions: This study provides a comprehensive evaluation of AI-based methods in SZ prognosis, emphasizing the strengths and limitations of current approaches. By identifying unresolved gaps, it offers valuable directions for future research in the application of AI for SZ detection and diagnosis.

## 1. Introduction

Schizophrenia (SZ), being a psychiatric condition, is noted for its deficits and distortions in feelings, behavior, thought, communication, and cognitive functions [1,2,3,4]. It is a psychological condition which impacts approximately 20 million individuals globally. It involves aberrant brain growth, resulting in symptoms such as paranoia and hearing unusual voices [5,6]. Individuals with SZ are more prone to experiencing premature death compared to healthy individuals, owing to the high prevalence of medical conditions. SZ impacts both genders, although evidence shows that males might develop the condition at an earlier age. Experts are investigating genetics and modern tools to understand the brain’s structure [7,8,9,10].

A competent psychiatrist diagnoses SZ based on the signs and diagnostic assessments to understand the individual’s background and state of mind [11]. New antipsychotic medications have not improved recovery rates, indicating that their efficacy remains stable [12]. Neuroimaging can aid clinicians in early illness diagnosis [13]. Recent research has used neuroscience and neuroimaging approaches, along with ML and DL, to automate the diagnosis of SZ [14,15,16].

**Figure 1:**
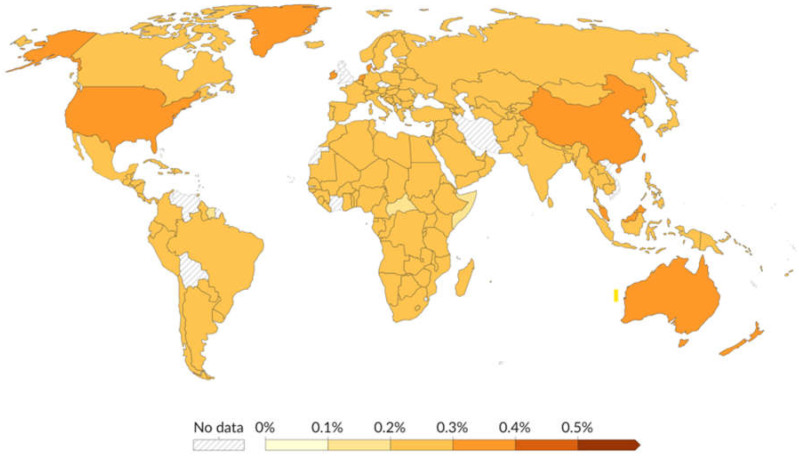
The projected rate of schizophrenia among a population of 100 individuals, adjusted for age, in the year 2021 (taken from [17]).

The world map in Figure 1 depicts the global burden of schizophrenia in 2021, highlighting the necessity for focused healthcare efforts in areas with greater prevalence rates [17]. The color gradient, which runs from bright yellow to dark orange, indicates the rising prevalence of schizophrenia, with deeper colors signifying higher rates. Many nations, including the majority of countries in Africa, South America, Europe, and Asia, have prevalence rates of 0.2% to 0.3%. Countries such as Australia, China, and the United States have a prevalence of 0.3% to 0.4%. India is tinted in mild orange, indicating a prevalence rate of 0.2% to 0.3%.

There are two types of neuroimaging studies used for diagnosing SZ: structural and functional [18]. Neuroimaging can help detect and diagnose psychotic illnesses, which are caused by aberrant brain structure and function [19]. Neuroimaging of brain structure comprises structural magnetic resonance imaging (sMRI) and diffusion tensor imaging (DTI). The investigations examine brain tissues (gray matter (GM), white matter (WM), and cerebrospinal fluid (CSF)) to identify abnormalities. However, the imaging of brain activity comprises electroencephalography (EEG) [20], magnetoencephalography (MEG) [21], functional magnetic resonance imaging (fMRI) [22], and functional near-infrared spectroscopy (fNIRS) [23].

EEG signals can detect and record minute changes in electrical activity in the brain, revealing important information about brain function [24]. EEG is less costly than are other neuroimaging procedures such as MRI or positron emission tomography (PET). EEG is easily accessible and non-invasive. EEG has high temporal resolution, collecting brain activity in real time [1,4]. EEG is more practicable for clinical usage. EEG signals can be accessed from high-quality open databases. EEG data can be used to detect SZ automatically using ML techniques. EEG has the ability to reveal underlying neurophysiological abnormalities linked with SZ. However, interpreting EEG data is difficult due to its large dimensionality and the delicate nature of the disorder-related signal alterations [25,26,27].

An sMRI scan assesses the brain’s anatomical features by analyzing its structure and connectivity. sMRI scans can reveal brain injury and abnormalities. They analyze GM and WM volume, as well as brain structure size, integrity, and shape [28]. fMRI is another commonly utilized technique for diagnosing SZ. fMRI records variations in the flow of blood to identify the regions of the brain responsible for vital processes. This approach operates on the principle that active brain areas experience increased blood flow. [29]. SZ is primarily caused by structural and functional brain abnormalities according to research [30]. SZ is associated with larger ventricles caused by GM deficiency [31]. Research suggests that cortical thickness decreases throughout normal childhood brain development and continues to decrease throughout adolescence and age [32].

**Figure 2:**
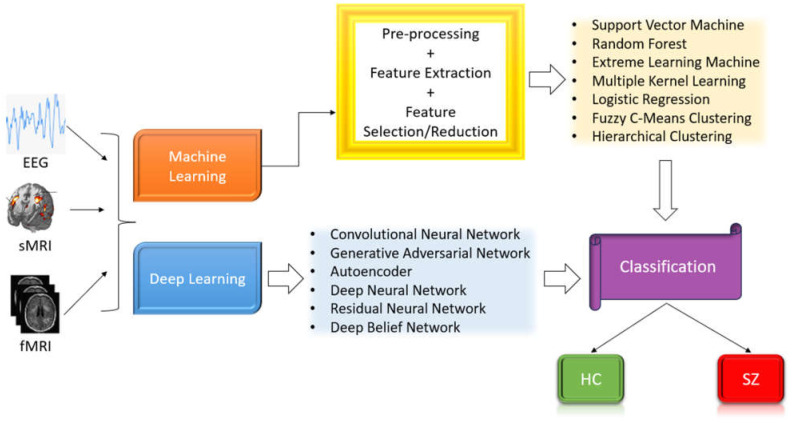
SZ diagnosis utilizing ML and DL techniques.

AI research focuses on creating intelligent machines capable of solving complex issues [33,34,35]. ML, a subset of AI, addresses prognosis, categorization, and modeling systems [36,37,38]. DL is a specific branch within the broader field of ML which collects information from big datasets, mimicking the human brain. During the last decade, high-performing classifiers were constructed for SZ diagnosis, with significant success [39]. DL models, particularly those based on convolutional neural networks (CNNs), are increasingly being used for extracting features and recognizing patterns of EEG data. These models have demonstrated outstanding ability to discover complicated patterns in huge datasets, making them ideal for biomedical applications [40,41,42]. Figure 2 illustrates the categorization of SZ and healthy controls (HC) utilizing ML and DL techniques applied to various imaging modalities.

### Motivation of the Present Survey

SZ is a complicated mental condition that necessitates interdisciplinary research involving neuroscience, psychology, psychiatry, and data science. This study’s goal was to synthesize findings from these several domains to provide a comprehensive picture of the advances in this sector. Over the past decade, there have been substantial technological developments and increased use of neuroimaging methods. This research helps us to understand how these advancements have helped to improve the diagnosis and classification of SZ. The survey aimed to assess various neuroimaging techniques (EEG, sMRI, and fMRI) in terms of efficacy, accuracy, and therapeutic applicability. This comparison can aid in finding the most successful approaches for various areas of SZ diagnosis. This work also identified gaps in current research and approaches by examining the existing literature. This can help to shape future research areas and increase the efficacy of SZ detection and categorization. Table 1 compares our survey study to other significant surveys on SZ diagnosis, including their limitations. We developed a new survey to address the research gaps identified in Table 1. The survey’s key contributions are noted below.

1. This survey includes all significant research papers published on SZ diagnosis between the year 2015 and 2024;
2. It provides an overview of SZ diagnostic methods, including ML and DL approaches;
3. It includes commonly utilized SZ datasets for both detection and classification purposes;
4. It also provides a critical overview of the existing approaches for SZ diagnosis;
5. The article concludes by analyzing future research opportunities in the topic.

**Table 1:** A basic review of prior surveys on SZ diagnosis and the present survey.

Work	Dataset Description	EEG Modality	MRI Modality	ML Techniques	DL Techniques	Detailed Critical Analysis	Drawbacks
Rahul et al. [43]—2024		✔		✔	✔		Dataset description is absent. Does not cover MRI-based publications. Does not provide detailed critical review.
Ranjan et al. [25]—2024	✔	✔			✔	✔	Does not cover MRI-based publications. ML-based approaches are not discussed.
Voineskos et al. [44]—2024			✔	✔		✔	Dataset description is absent. Does not cover EEG-based publications. DL-based approaches are not discussed.
Jafari et al. [45]—2023	✔	✔		✔	✔	✔	Does not cover MRI-based publications.
Verma et al. [46]—2023	✔	✔	✔	✔	✔		Dataset description is brief. Does not provide detailed critical review.
J.A. Cortes-Briones et al. [47]—2022		✔	✔		✔		Dataset description is absent. ML-based approaches are not discussed. Does not provide detailed critical review.
Sadeghi et al. [48]—2022			✔	✔	✔	✔	Dataset description is absent. Does not cover EEG-based publications.
Barros et al. [49]—2021		✔		✔	✔	✔	Dataset description is absent. Does not cover MRI-based publications.
Luján et al. [50]—2021		✔		✔			Dataset description is absent. Does not cover MRI-based publications. DL-based approaches are not discussed. Does not provide detailed critical review.
Lai et al. [51]—2021		✔	✔	✔			Dataset description is absent. DL-based approaches are not discussed. Does not provide detailed critical review.
Steardo Jr. et al. [52]—2020			✔	✔			Dataset description is absent. Does not cover EEG-based publications. DL-based approaches are not discussed. Does not provide detailed critical review.
de Filippis et al. [53]—2019			✔	✔			Dataset description is absent. Does not cover EEG-based publications. DL-based approaches are not discussed. Does not provide detailed critical review.
Proposed Survey—2024	✔	✔	✔	✔	✔	✔	-

**Figure 3:**
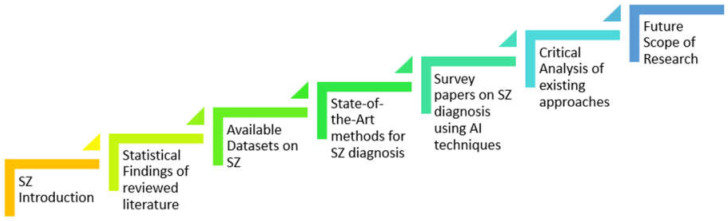
Flow chart of the proposed survey on the automatic detection of SZ.

The following portions of the paper are arranged as follows: Section 2 provides the statistical findings of our reviewed literature. Section 3 provides the publicly available datasets. Section 4 provides the state-of-the-art methods along with all major research publications between the year 2015 and 2024. Section 5 outlines a critical review of existing approaches. Section 6 concludes the paper and outlines the research prospects. Figure 3 illustrates our survey’s brief workflow.

## 2. Materials and Methods

**Figure 4:**
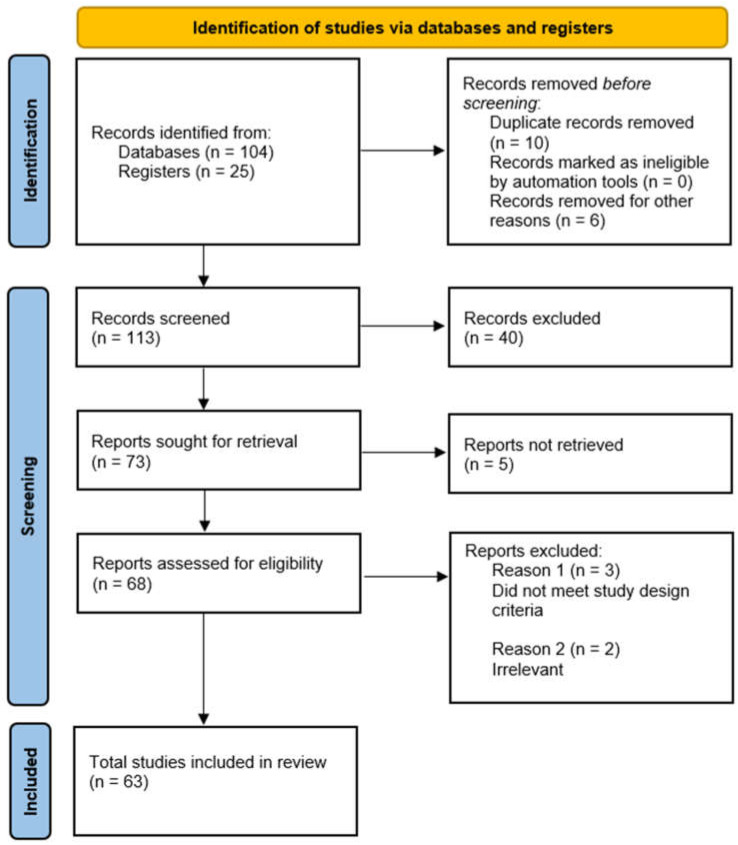
PRISMA 2020 flow diagram for selecting appropriate literature on the automatic detection of SZ.

A systematic review of research papers was conducted using key terms such as “Schizophrenia”, “Artificial Intelligence”, “Machine Learning”, “Deep Learning”, “EEG”, “CWT”, “sMRI”, “fMRI”, and their variants. The literature was assessed for relevance before inclusion in the review. This systematic review was conducted in accordance with the PRISMA (Preferred Reporting Items for Systematic Reviews and Meta-Analyses) 2020 guidelines to ensure transparent and comprehensive reporting of the review process and findings. We used the PRISMA 2020 checklist [54] when writing our report, which is provided in the Supplementary Materials along with the manuscript. The review protocol was registered in the International Prospective Register of Systematic Reviews (PROSPERO) [55] under registration number: CRD42024612364. The flow diagram in Figure 4 illustrates the systematic screening process. A total of 129 records were initially identified from databases (104 records) and registers (25 records). Sixteen records were removed before screening. This included 10 duplicate records and 6 records removed for other reasons. After the initial removal, 113 records were screened for relevance to the review topic. Forty records were excluded based on predefined criteria at this screening stage. From the screened records, 73 reports were identified for further retrieval. Five reports could not be retrieved due to accessibility issues. Sixty-eight reports were evaluated in more detail for eligibility. Five reports were excluded at this stage, with the reasons being that these studies did not meet the required study design criteria (*n* = 3) and that these studies were deemed irrelevant to the research question (*n* = 2). After this process, a total of 63 studies met all criteria and were included in the final review.

**Figure 5:**
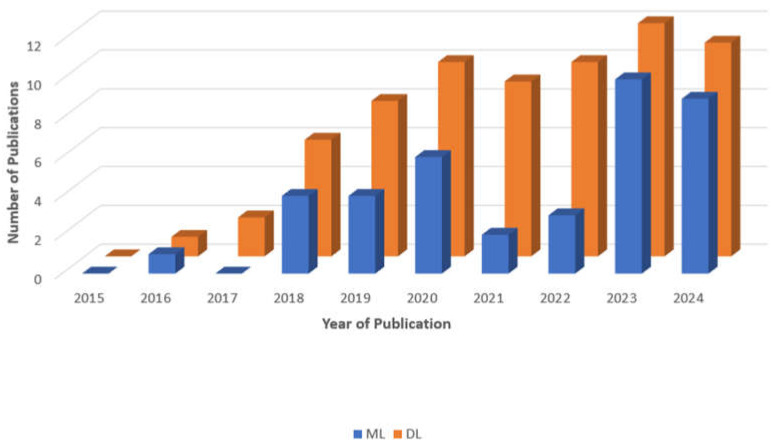
A bar graph depicting the number of articles related to the diagnosis of SZ with ML and DL methodologies published during the past decade. This is a non-exhaustive list of publications.

This study aimed to investigate AI strategies for SZ diagnosis utilizing EEG, sMRI, and fMRI. This survey will provide SZ scientists with information on the latest breakthroughs relevant to the diagnosis of SZ. Figure 5 displays the number of publications (both journals and conferences) on SZ diagnosis with ML or DL approaches published from 2015 to 2024. Out of 108 research publications (which may not be a comprehensive list), 39 were based on ML methods and 69 were based on DL models.

## 3. Available Datasets

This section discusses the openly accessible datasets for SZ. The SZ dataset includes neuroimaging modalities used to detect the condition. EEG, sMRI, and fMRI are commonly utilized modalities for SZ research. Openly accessible datasets relevant to SZ diagnosis include COBRE [56], RepOD [57], NUSDAST [58], UCLA [59], SchizConnect [60], MCIC [61], MLSP2014 [62], MSU [63], and FBIRN [64], as described further. Table 2 provides a summary of all the freely available datasets used for SZ diagnosis.

**Table 2:** Particulars of publicly accessible datasets for SZ diagnosis.

Dataset	Publisher	Modality	Number of Samples	Sample Diversity	Data Collection Biases	Download Link
COBRE [56]—2012	Mind Research Network and the University of New Mexico	sMRI and fMRI	SZ = 72 HC = 75	Primarily U.S.-based, limited demographic diversity (age 18–65)	Demographic homogeneity, exclusion of comorbid conditions, institutional differences	http://fcon_1000.projects.nitrc.org/indi/retro/cobre.html (accessed on 20 July 2024)
RepOD [57]—2017	Institute of Psychiatry and Neurology in Warsaw, Poland	EEG	SZ = 14 HC = 14	Focused on paranoid schizophrenia subtype	Exclusion of other psychiatric disorders	https://repod.icm.edu.pl/dataset.xhtml?persistentId=doi:10.18150/repod.0107441 (accessed on 15 September 2023)
NUSDAST [58]—2015	National University of Singapore (NUS)	sMRI	SZ = 171 HC = 170 Strict SZ = 44 No disorder = 66	Mixed demographics	Potential institutional bias, overrepresentation of certain clinical subtypes	https://sites.wustl.edu/oasisbrains/central-xnat/ (accessed on 22 July 2024)
UCLA [59]—2013	OpenfMRI project	sMRI and fMRI and DWI	SZ = 50 HC = 130 Patients with ADHD = 43 Bipolar illness = 49	Diverse group covering SZ, bipolar disorder, ADHD, and HC	Possible overrepresentation of specific disorders (SZ, bipolar), lack of detailed demographic data.	https://openfmri.org/dataset/ds000030/ (accessed on 22 July 2024)
SchizConnect [60]—2015	SchizConnect consortium	sMRI and fMRI	No disorder = 632 Strict SZ = 384 Broad SZ = 215 Bipolar disorder = 10 Schizoaffective disorder = 41 Siblings of unknown disorder = 66 Siblings of strict SZ = 44	Diverse diagnostic groups, limited demographic details	Overrepresentation of certain diagnostic groups (strict SZ, broad SZ), potential selection bias from varied data sources	http://www.schizconnect.org/ (accessed on 22 July 2024)
MCIC [61]—2013	Mind Research Network (MRN)	sMRI and fMRI and DWI	SZ = 162 HC = 169	Wide age range, limited to specific geographic and clinical settings	Potential bias due to geographic focus	https://www.nitrc.org/projects/mcic/ (accessed on 20 July 2024)
MLSP2014 [62]—2014	IEEE	sMRI and fMRI	SZ = 69 HC = 75	Limited demographic variation	Potential bias from focus on structural and functional brain features	https://www.kaggle.com/c/mlsp-2014-mri (accessed on 20 July 2024)
MSU [63]—2017	Lomonosov Moscow State University	EEG	SZ = 45 HC = 39	Specific to age group, limited broader representation	Age-specific focus (adolescents)	http://brain.bio.msu.ru/eeg_schizophrenia.htm (accessed on 15 September 2023)
Phase II of FBIRN [64]—2007	FBIRN consortium	fMRI	SZ = 87 HC = 85	Moderate diversity, spanning ages 18–70 years, limited geographic representation	Potential overrepresentation of specific SZ subtypes, age-related bias	https://www.ncbi.nlm.nih.gov/pmc/articles/PMC4651841/ (accessed on 23 July 2024)
Phase III of FBIRN [64]—2007			SZ = 176 HC = 186	Broader sample with similar age range (18–70 years), limited demographic diversity	Potential selection bias due to specific inclusion criteria	

### 3.1. COBRE

**Figure 6:**
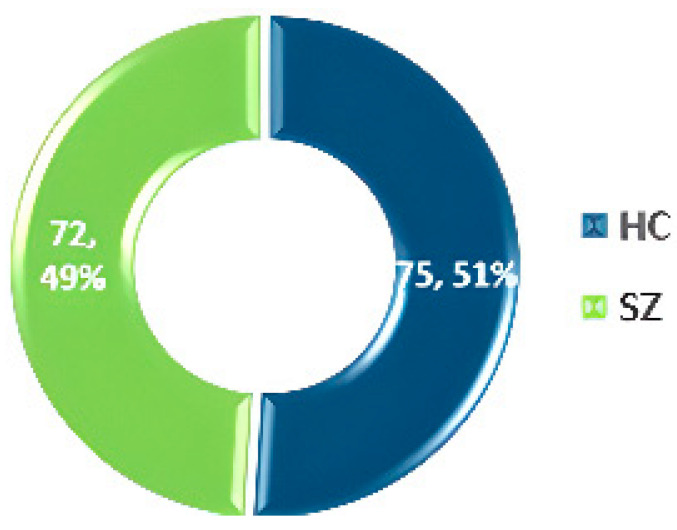
Data statistics of the COBRE dataset [55].

The Center for Biomedical Research Excellence (COBRE) [56] conducted fMRI on 147 volunteers, comprising 72 SZ patients and 75 HC aged 18–65. The dataset includes phenotypic details such as handedness, age, diagnosis, and gender. The dataset statistics are shown in Figure 6.

### 3.2. RepOD

**Figure 7:**
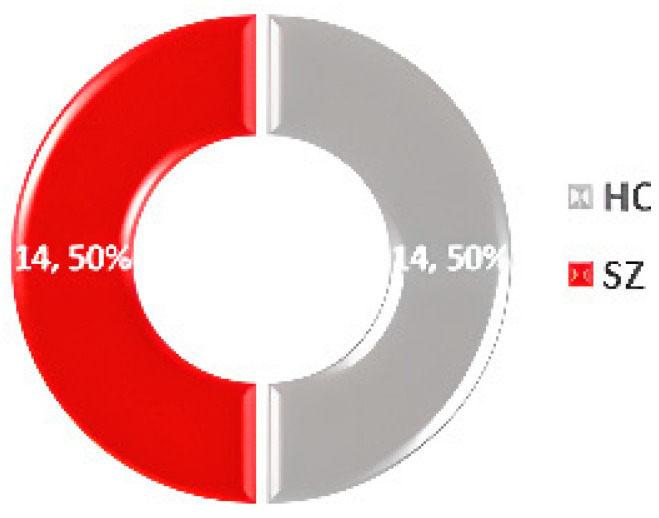
Data statistics of the RepOD dataset [57].

The Repository for Open Data (RepOD) [57] offers an EEG dataset from Olejarczyk and Jernajczyk’s study [65], containing data from 14 paranoid SZ patients and 14 HC, including 14 men and 14 women aged 18 or older. The data were collected using 19 EEG channels with a sampling frequency of 250 Hz. The dataset statistics are shown in Figure 7.

### 3.3. NUSDAST

**Figure 8:**
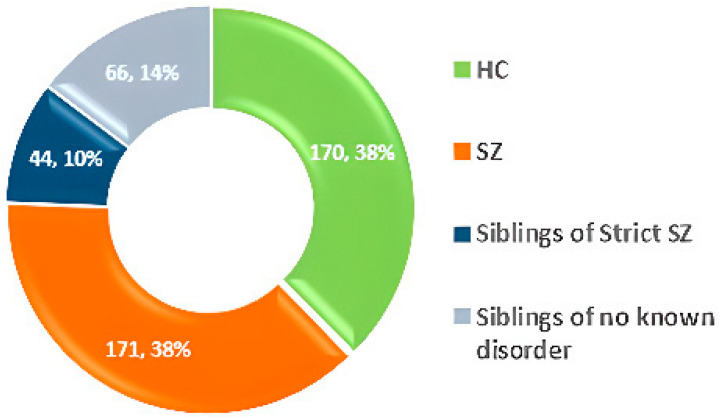
Data statistics of the NUSDAST dataset [58].

The Northwestern University Schizophrenia Data and Software Tool (NUSDAST) [58] includes MRI brain imaging data from over 450 participants, along with medical, hereditary, and psychological information. Dataset statistics are shown in Figure 8.

### 3.4. UCLA

**Figure 9:**
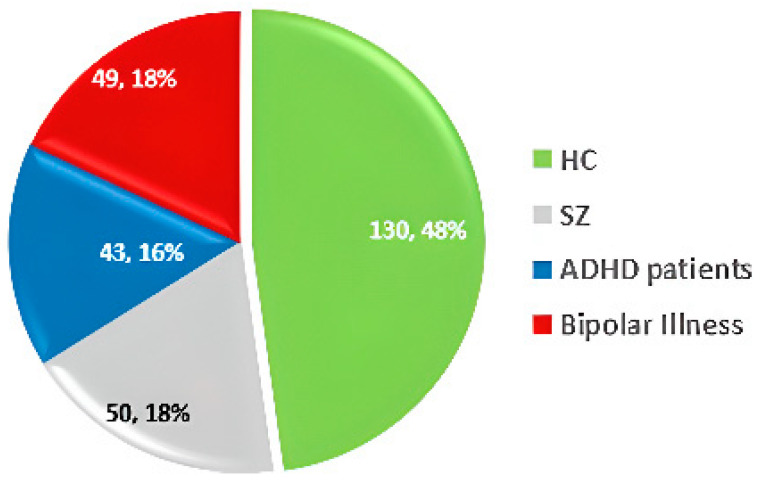
Data statistics of the UCLA dataset [59].

The UCLA dataset [59] includes sMRI, fMRI, and Diffusion-weighted imaging (DWI) data, along with phenotypic information. It comprises 50 SZ patients, 130 HC, 43 ADHD patients, and 49 individuals with bipolar disorder. Dataset statistics are shown in Figure 9.

### 3.5. SchizConnect

**Figure 10:**
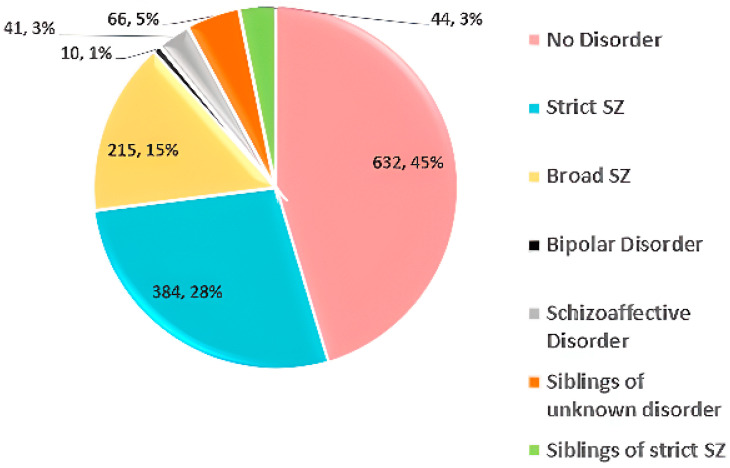
Data statistics of the SchizConnect dataset [60].

SchizConnect [60] is a data repository combining brain imaging data relevant to schizophrenia from various sources. It includes 1392 subjects: 632 HC, 384 with strict SZ, 215 with broad SZ, 10 with bipolar disorder, 41 with schizoaffective disorder, 66 siblings with unknown disorders, and 44 siblings with strict SZ. Dataset statistics are shown in Figure 10.

### 3.6. MCIC

**Figure 11:**
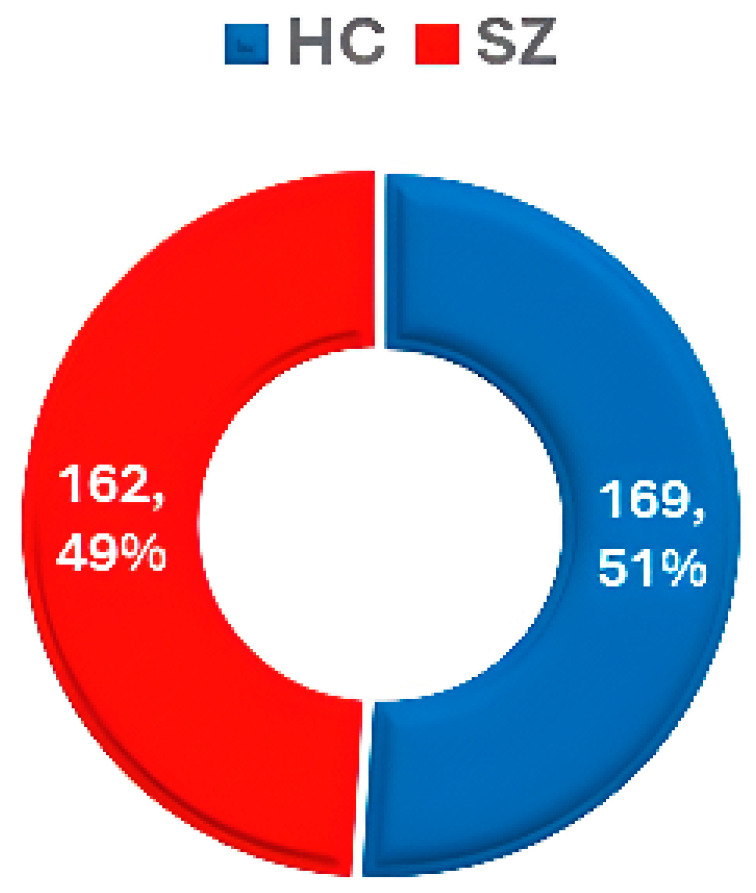
Data statistics of the MCIC dataset [61].

The MCIC dataset [61] includes data from 162 SZ patients and 169 HC, along with demographic features such as sex and age group. Dataset statistics are shown in Figure 11.

### 3.7. MLSP2014

**Figure 12:**
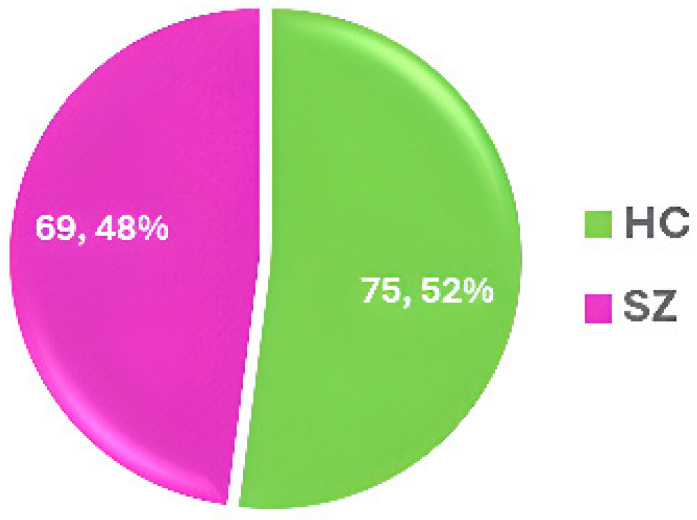
Data statistics of the MLSP2014 dataset [62].

The MLSP 2014 competition [62] used a dataset that includes structural morphometry and functional connectivity data from MRI scans of 69 SZ patients and 75 HCs. The dataset statistics are shown in Figure 12.

### 3.8. MSU

**Figure 13:**
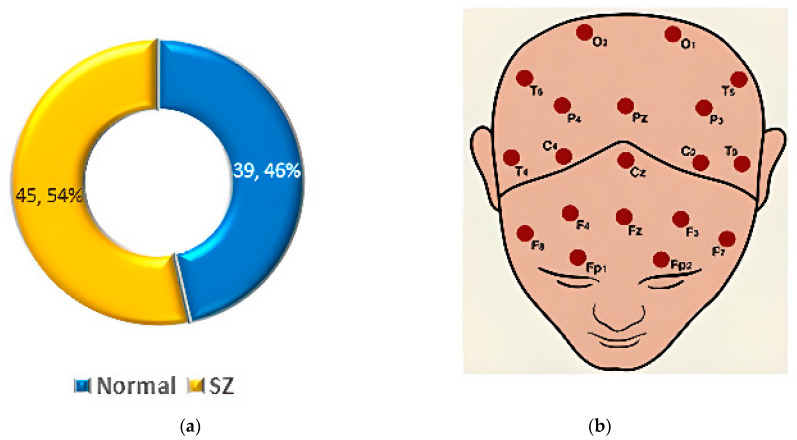
(**a**) Data statistics of the MSU EEG dataset. (**b**) Topographical positions of channel numbers [63].

The dataset from M.V. Lomonosov Moscow State University (MSU) [63] includes EEG recordings from adolescents, divided into two groups: 39 HCs and 45 showing symptoms of SZ. The dataset statistics are shown in Figure 13a.

Each EEG file contains recordings from 16 channels, with EEG amplitudes (in µV) captured at a sampling rate of 128 Hz. Each file consists of 7680 samples per channel, corresponding to 1 min of data. The channels are T6, F3, O1, Pz, C4, T5, Cz, F8, P4, O2, P3, T3, F7, C3, T4, and F4, as shown in Figure 13b [63].

### 3.9. FBIRN

**Figure 14:**
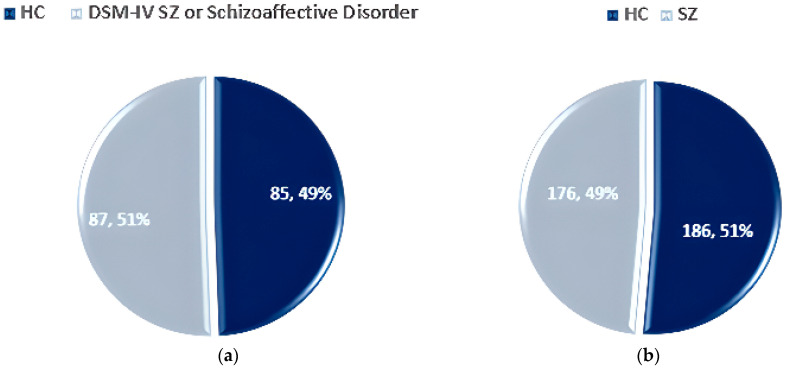
Data statistics of the FBIRN dataset: (**a**) Phase II and (**b**) Phase III [64].

The FBIRN dataset [64] consists of three phases, with the 2nd and 3rd phases containing data on SZ individuals. Phase 2 includes 87 SZ patients and 85 HCs, aged 18–70 years. Phase 3 contains 176 SZ patients and 186 HCs. The dataset statistics for Phase II and Phase III are shown in Figure 14a and Figure 14b respectively.

## 4. Cutting-Edge Approaches and Results of SZ Diagnosis

**Figure 15:**
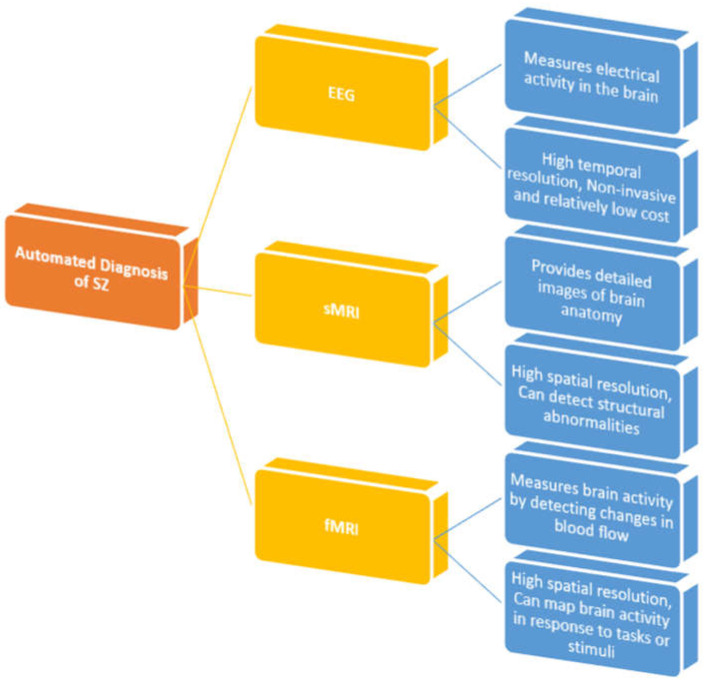
Taxonomy of neuroimaging modalities for SZ detection and classification.

Several researches have employed AI to detect SZ utilizing EEG, sMRI, and fMRI modalities. Section 4.1, Section 4.2 and Section 4.3 cover the ML and DL approaches and the consequent outcomes of SZ diagnosis. Figure 15 shows the imaging techniques of the brain for automated SZ diagnosis.

### 4.1. EEG-Based Diagnosis of SZ

**Figure 16:**
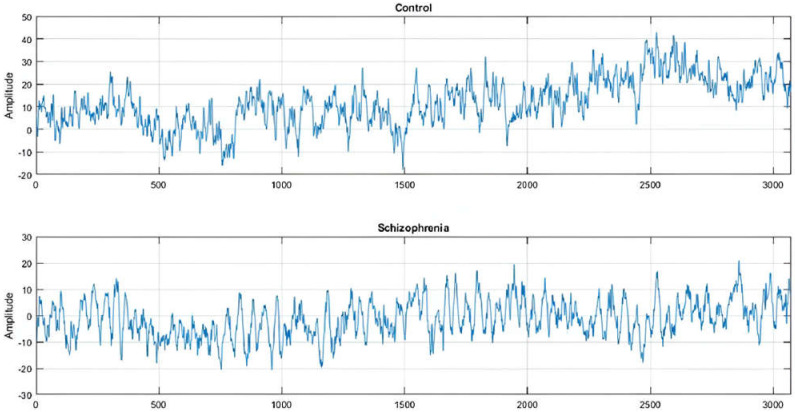
EEG signals of an HC and an SZ patient (obtained from [67]).

EEG signals have been shown to be effective in detecting SZ in clinical settings. EEG is a cost-effective and high-resolution diagnostic technique for a range of diseases [66]. CAD systems aid in the reliable diagnosis of SZ. ML approaches employing EEG signals have become increasingly popular for diagnosing mental diseases. Researchers have used DL models, such as CNN and LSTM, to improve SZ diagnosis. Figure 16 displays the EEG waveform obtained from the F7 electrode to distinguish between an SZ and HC individual [67]. The imbalance in wave amplitude is steady across the measurement. Lower amplitude signals are known to be aberrant or to indicate a psychotic condition.

In recent years, there has been substantial research on detecting SZ using EEG signals. There are many datasets available publicly.

Using the Kaggle EEG dataset [68], Lei Zhang [69] developed an artificial neural network (ANN) design where EEG signals were recorded using event-related potential (ERP) corresponding to button press and audio tone playback. Five temporal features taken from EEG data, as well as two demographic features, were utilized to train and test the ANN. Following the wavelet transform of the ERP EEG signals for ANN classification, additional time–frequency features were recovered. The highest classification accuracy of more than 98.5% was achieved. These research findings indicate a significant potential for developing an accurate and subjective diagnosis tool for SZ based on EEG signals. Siuly et al. [70] investigated the use of deep residual networks (ResNet) to automatically detect SZ from EEG signals. The process included three major stages: the preprocessing of EEG signals using the average filtering approach to reduce noise, feature extraction where a deep ResNet was used to automatically extract important features from EEG recordings, and the classification of retrieved features via a Softmax layer. In addition, multiple ML approaches, including a support vector machine (SVM), were applied to the same feature set. Their proposed method achieved 99.23% accuracy with the SVM classifier, outperforming the ResNet classifier and other existing approaches. C. Barros [71] demonstrated a DL-based technique for automatically detecting SZ using EEG signals. This research highlights the effectiveness of DL approaches in exploring impaired auditory processing in SZ, with potential diagnostic implications.

Using the RepOD dataset [57], Buettner et al. [72] developed a rapid, high-performance classification approach for diagnosing SZ. The model primarily comprised three preprocessing steps: independent component analysis (ICA), spectral analysis utilizing Buettner et al.’s 99-frequency-band approach, and normalization. The random forest (RF) approach was employed for classification. The model could exclude SZ with 100% accuracy. This approach, when combined with a differential diagnosis system, can speed up, improve the accuracy, and reduce the cost of ICU treatments. Krishnan et al. [73] focused on multivariate empirical mode decomposition (MEMD) and entropy measures to detect SZ. The researchers employed MEMD to break down EEG signals into intrinsic mode functions (IMF) signals. Computing the signal’s entropy yielded the randomness measure for the IMF signal. Numerous ML classifiers were trained using a feature matrix obtained from the entropy values of the IMF signal. Among these, SVM with a radial basis function (SVM-RBF) achieved the highest accuracy and F1-score of 93% for the 95 features. It also produced an AUC of 0.9831. Shoeibi et al. [74] investigated the early identification of SZ using EEG signals and a one-dimensional transformer model. EEG signals were pre-processed by filtering, normalizing, and segmenting into time frames. Feature extraction entailed using a one-dimensional transformer architecture with various activation functions to extract features from pre-processed EEG signals. The Softmax classifier was used in the final stage. The proposed model had a maximum accuracy of 97.62% in diagnosing SZ, indicating its potential efficacy. Sara et al. [75] demonstrated a unique strategy where the EEG data were analyzed using the transfer entropy (TE) method to determine the effective connection matrix. The hybrid framework of pre-trained CNN-LSTM models outperformed the pre-trained CNN models. The EfficientNetB0-LSTM model yielded the highest average accuracy and F1-score via the 10-fold cross-validation procedure, at 99.90% and 99.93%, respectively.

Using a clinical dataset [76], Taylor et al. [77] conducted a study aimed at developing an objective, biologically based computational tool for diagnosing SZ using EEG. The subjects were exposed to three auditory oddball paradigms, which consisted of tonal sequences that varied from standard tones in 10% of the trials. The authors employed multivariate pattern analysis and identified the snapshots created using statistical parametric mapping (SPM) of the spatiotemporal EEG data, specifically ERPs recorded on the 2D surface of the scalp. For classification, they used SVM and Gaussian process classifiers (GPCs). The classification of individual patients and controls achieved an accuracy as high as 80.48% (*p*-value = 0.0326, adjusted for the false discovery rate). Receiver operating characteristic (ROC) analysis produced an area under the curve (AUC) of 0.87. Gaussian process regression analysis demonstrated that the mismatch negativity predicted GAF scores, with a correlation of 0.73, an R2 of 0.53, and a *p*-value of 0.0006. Chang et al. [78] examined functional connectivity in the brain during the mismatch negativity procedure in HC and SZ participants. The researchers used accurate low-resolution electromagnetic tomography to reconstruct cortical endogenous electrical activity from EEG recordings, and they established functionality of brain structures utilizing EEG. They retrieved graph theory properties of brain structures and classified FESZ, CSZ, and HC groups with an SVM. They also proposed a graph neural network (GNN) architecture that can learn directly from the brain structure. This study discovered that CSZ involves more damaged brain areas than does FESZ, with the auditory cortex being particularly harmed. This highlights the variety of SZ’s effects across different illness histories, as well as the relationship between the MMN and the auditory cortex. The GNN classifier trained on brain functional networks had an accuracy of 84.17%. This considerably outperformed an SVM classifier trained on graph-theoretic characteristics, which had a maximum accuracy of 69.17%. Febles [79] described a study that used ML to diagnose SZ. Using the multiple kernel Learning (MKL) classifier and the entire dataset, the study achieved an 83% classification accuracy. After the Boruta feature selection technique was applied, the classification accuracy increased to 86%. The auditory P300 paradigm’s latency and amplitude were the most important criteria in the classification.

**Figure 17:**
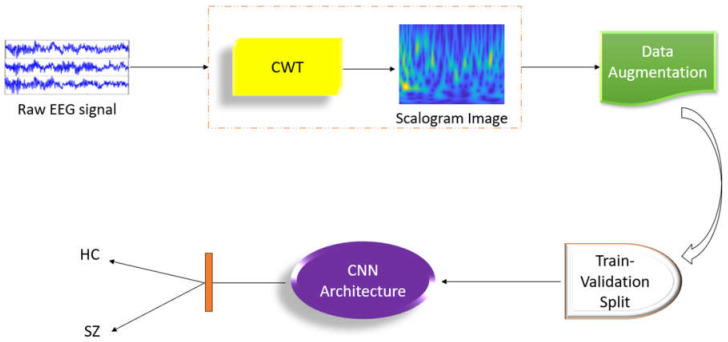
Flowchart of the CAD approach for automatic SZ detection with EEG signals developed by Aslan et al. [1].

Using the dataset from the Mental Health Research Center [63] and the Institute of Psychiatry and Neurology in Warsaw [57], Aslan et al. [1] conducted a study that used DL techniques to diagnose SZ. The EEG signals were transformed to 2D using the continuous wavelet transform (CWT) technique for acquiring time-frequency characteristics. Unlike typical ML techniques, the network’s training features were automatically retrieved from EEG recordings. The research successfully classified SZ patients and healthy persons with 98% and 99.5% accuracy, respectively, using two separate datasets containing individuals of various ages. The interpretability of the results, as visualized using several methodologies, clearly indicated a relationship between spectral elements in HCs and SZ patients. The computer-aided diagnostic (CAD) approach for automatic SZ detection utilizing EEG signals is shown in Figure 17. Saadatinia et al. [80] developed an approach for automatic SZ diagnosis based on EEG brain recordings. Spectrograms were created from raw EEG signals. The original diagnosis was made using a convolutional neural network (CNN). The variational autoencoder-augmented dataset yielded a 3.0% increase in accuracy, reaching 99.0%. The authors also utilized the local interpretable model-agnostic explanations (LIME) algorithm to increase trust in the diagnostic process.

Using the MSU dataset [63], C. Phang et al. [81] completed a study that employed a deep neural network with deep belief network (DNN-DBN) architecture for SZ classification using EEG. Their approach attained a 95% classification accuracy in the theta and beta bands. Rajesh et al. [82] described a study that used symmetrically weighted local binary patterns (SLBPs) for SZ identification. The authors proposed an SLBP-based automated method for detecting SZ. The SLBP-based histogram characteristics were passed via a correlation-based feature selection technique. Finally, the feature vector produced was fed into a LogitBoost classifier to distinguish patients with SZ from HCs. The suggested approach attained an accuracy of 91.66%. Sobahi et al. [83] described a study that employed a unique approach for detecting SZ via EEG recordings. The authors proposed a novel signal-to-image mapping approach. The obtained rhythm signals from EEG recordings were encoded using the 1D local binary pattern (LBP). During the data augmentation step, extreme-learning-machine (ELM)-based autoencoders (AE) were used. Notable deep transfer learning methods were used to distinguish patients with SZ from HCs. Their proposed approach achieved a 97.7% accuracy.

Table 3 provides a table summarizing the study characteristics of the key research on employing EEG signals for SZ identification.

**Table 3:** Study characteristics of notable research relevant to SZ diagnosis utilizing EEG (patients—with SZ).

Work	Dataset	Number of Samples	Data Preparation	Software for Data Preparation	Feature Extraction Method	Approach	Result (%)
Lei Zhang [69]—2020	Kaggle Basic Sensory Task data	Patients = 49, controls = 32	Baseline selection, min–max normalization	-	Temporal, spatial, demographic & time–frequency features	Artificial neural network	Accuracy = 98.5
Siuly et al. [70]—2023		Patients = 49, controls = 32	Average filtering	-	Deep ResNet	Softmax Layer and deep features with SVM	Accuracy = 99.23
Buettner et al. [72]—2019	RepOD	Patients = 14, controls = 14	ICA, normalization	-	Fourier transformation	Random Forest	Accuracy = 100
Krishnan et al. [73]—2020		Patients = 14, controls = 14	-	-	Extraction using MEMD and entropy measures	SVM-RBF	Accuracy = 93, precision = 92, recall = 94
Shoeibi et al. [74]—2024		Patients = 14, controls = 14	Filtering, normalization, segmentation into time windows	-	1D transformer architecture	Softmax classifier, 10-fold cross-validation	Accuracy = 97.62
Sara et al. [75]—2022	RepOD-IBIB PAN	Patients = 14, controls = 14	-	-	Connectivity matrix, TE	CNN-LSTM, 10-fold cross validation	Accuracy = 99.9
Febles [79]—2022	Clinical	Patients = 54, controls = 54	Filtering, baseline correction, artifact rejection	-	Features related to peak-to-peak measurements and signal characteristics, Boruta algorithm	Multiple kernel learning	Accuracy = 86
Aslan et al. [1]—2022	Mental Health Research Center, Institute of Psychiatry & Neurology in Warsaw	Patients = 45 healthy = 39 Patients = 14, controls = 14	-	-	Time–frequency features	CNN	Accuracy = 98, precision = 98, recall = 98 Accuracy = 99.5, precision = 99, recall = 99
Saadatinia et al. [80]—2024		Patients = 45, controls = 39 Patients = 14, controls = 14	-	-	-	CNN, WGAN-GP and VAE	Accuracy = 99
C. Phang et al. [81]—2019	MSU	Patients = 45, controls = 39	-	-	DC-CN features	DNN-DBN	Accuracy = 95
Rajesh et al. [82]—2021		Patients = 45, controls = 39	-	-	SLBP-based histogram features	LogitBoost Classifier	Accuracy = 91.66
Sobahi et al. [83]—2022		Patients = 45, controls = 39	-	-	Time–frequency features	ELM-based AE	Accuracy = 97.7

### 4.2. sMRI-Based Diagnosis of SZ

**Figure 18:**
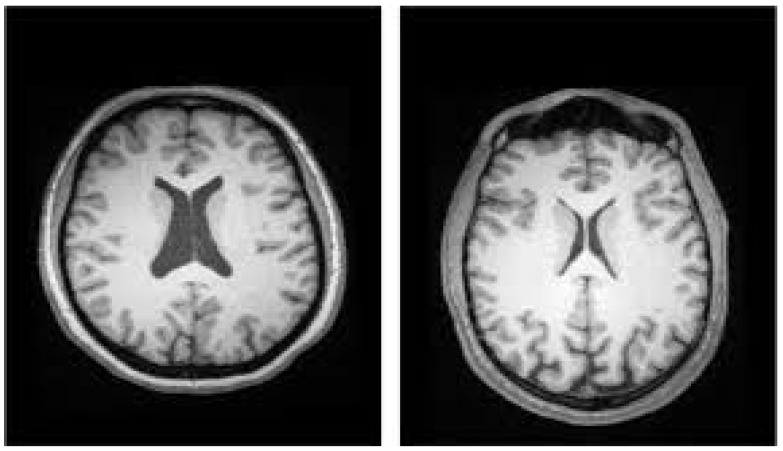
SZ diagnosis utilizing sMRI ((**Left**): enlarged ventricles indicate SZ. (**Right**): typical ventricles indicate a healthy condition) [29].

Several studies have used ML techniques to diagnose SZ utilizing sMRI modalities. Many researchers have used logistic regression (LR), RF, or SVM classifiers to classify data at the subject level [84]. Figure 18 depicts an sMRI scan for distinguishing between the ventricles of HCs and SZ participants [29].

Using the COBRE dataset [56], Qureshi et al. [85] employed a combined weighted feature integration comprising neural networking of the brain as well as morphological characteristics extracted from MRI data, which was then analyzed using an extreme learning machine (ELM). This technique attained a maximum accuracy of 99.29%. In 2018, M. Latha et al. [86] conducted a study on structural biomarkers in the SZ brain using T1-weighted MRI data from the COBRE database. For segmentation, they used a neighborhood-clustering-based level set technique, and texture analysis was performed using Laws texture features. The approach revealed significant differences in geometric features between normal and SZ brains with texture features. Ramkiran et al. [87] investigated resting-state anticorrelated networks in SZ. To choose largely anticorrelated networks, the method entailed generating functional connectivity matrices and then applying the anticorrelation after mean of antilog (AMA) method. The study discovered anomalies in anticorrelated networks connecting subcortical and cortical locations. Chen et al. [88] used sMRI data from SZ patients and normal controls (NCs) to distinguish between the two groups. The method employed a ML framework with a coarse-to-fine feature selection methodology that included two-sample *t*-tests, recursive feature elimination (RFE), and SVM for classification. The framework focused on GM and WM properties and achieved a classification accuracy of more than 85%, allowing for individual diagnosis and correlating with earlier biomarker research. This procedure can potentially be used for other disorders as well. Guo et al. [89] investigated the application of ML to SZ categorization based on brain morphology. Morphological data from 26 hippocampal and 20 amygdaloid subregions were retrieved from T1 sMRI scans. For feature selection, the sequential backward elimination (SBE) approach was utilized, and classification was performed utilizing a linear SVM classifier. The SBE-SVM model’s classification accuracy was 81.75%, with a sensitivity of 84.21% and a specificity of 81.16%. The area under the curve (AUC) was 0.82411. The study shows how ML algorithms can be used to classify SZ based on subcortical morphological traits. Tanveer et al. [90] employed ML to improve the diagnosis of SZ. Several categorization techniques were tested, including SVM, RF, kernel ridge regression, and randomized neural networks. The *t*-Test, ROC, Wilcoxon, Entropy, Bhattacharyya, MRMR, and NCA were all used to select features. This study emphasizes the significance of classification algorithms in SZ diagnosis.

Using the NUSDAST dataset [58], Talpalaru et al. [91] examined SZ subgroups using ML. Clinical subgroups were identified by hierarchical clustering, and three ML models (LR, SVM, RF) were utilized for predicting subgroup membership based on cortical thickness estimates in 78 regions of interest. The RF model fared the best, with an AUC of 0.81 for the high-symptom-burden group and 0.78 for the moderate-symptom-burden group, outperforming the baseline comparison AUC of 0.75. Pinaya et al. [92] proposed a method that uses DL to identify aberrant brain areas in neuropsychiatric illnesses. A deep autoencoder, or artificial neural network, was used to quantify overall and regional neuroanatomical aberrations in individuals with SZ and autism spectrum disorder utilizing two independent datasets (*n* = 263). The model accurately distinguished between diseases and controls, producing discrete values of neuroanatomical deviation for each disease (*p* < 0.005), consistent with previous neuroimaging research. This work demonstrated the utility of deep autoencoders for detecting neuroanatomical abnormalities in neuropsychiatric populations.

Using a clinical dataset [76], Zarogianni et al. [93] investigated the use of a variety of data types to improve the prediction of SZ in high-risk patients. An SVM classifier with recursive feature elimination (RFE) was utilized in a nested cross-validation strategy to find relevant predictors and increase diagnostic accuracy. Rather than utilizing each measure alone, the model combined schizotypal measurements, a declarative memory test, and MRI data and achieved a classification accuracy of 94%. J. Liu et al. [94] proposed a strategy for classifying SZ using a variety of imaging techniques. The authors created a multi-modal, multi-atlas feature representation, and multi-kernel learning method (MMM). They retrieved eight feature sets from the MRI data using four brain atlases and four markers, followed by a two-step feature selection procedure. For SZ and HC categorization, the MMM technique achieved an accuracy of 91.28%, a sensitivity of 90.85%, a specificity of 92.17%, and an AUC of 0.9485. A.V. Nimkar et al. [95] sought to improve the prediction of SZ diagnosis by ML approaches. ML approaches were used to obtain the highest binary classification accuracy for predicting SZ. Nguyen H. et al. [96] addressed the issue of variability in multi-site neuroimaging data. Their study focused on T1-weighted brain pictures obtained from various locations. The authors employed generative adversarial networks (GANs) to alter photos from one site to match those from another, with the goal of reducing site-specific differences while retaining gender or clinical-diagnosis-relevant information. The usefulness of GANs in normalizing image sets to a common scanner set was tested, and the model was shown to perform effectively, with less information loss than that of contemporaneous techniques. This strategy enabled the pooling of neuroimaging data from various sites, increasing research sensitivity and statistical power without requiring thorough knowledge of the origins of bias. Srinivasagopalan et al. [97] reported on a DL approach for diagnosing SZ. The National Institute of Health [76] contributed the secondary dataset used in the study. The researchers compared classic ML methods such as LR, SVMs, and RFs against a DL model with three hidden layers. The DL model demonstrated better accuracy in diagnosing SZ, indicating that it could be a substantial development in the field.

Using the B-SNIP dataset [98], Rokham et al. [99] employed a multi-label data purification strategy with T1 sMRI data from 1493 people to detect label noise in the diagnoses of mood and psychotic disorders. The method comprised numerous classifications with an SVM and relabeling based on MRI data, and it was very accurate in identifying label noise.

**Figure 19:**
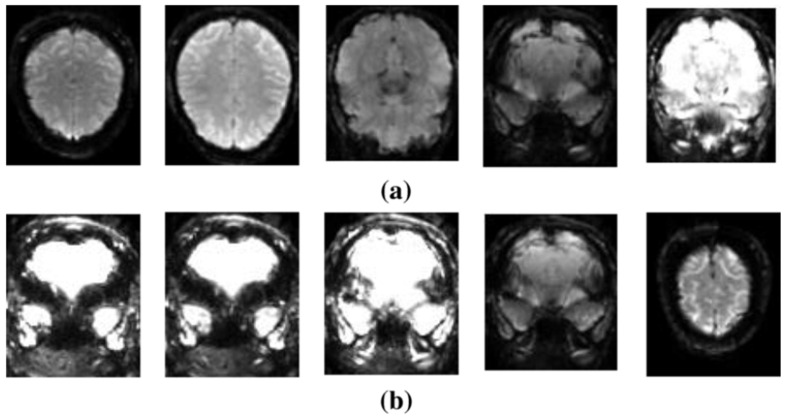
Snapshots of various categories within the NAMIC dataset: (**a**) HCs and (**b**) patients with SZ [104].

Using the SchizConnect dataset [60], Oh et al. [100] used a DL algorithm to assess sMRI data to diagnose SZ. The model performed well, with an AUC of 0.96 showing that it is useful in detecting SZ. Junhao et al. [101] used DL and 3D structural brain MRI data to detect SZ. Their model was trained and tested on three open datasets using conventional T1-weighted MRI scans, yielding an AUC of 0.987, suggesting nearly flawless accuracy in differentiating SZ patients from HCs. Using the NAMIC dataset [102], Manohar et al. [103] proposed a method for detecting SZ using MR brain scans, which attained an accuracy of 90% and an AUC of 0.9, suggesting its efficacy in discriminating between SZ and healthy individuals. Figure 19 displays the visuals of this dataset [104]. Latha et al. [105] used a metaheuristic algorithm and a radiomics technique to study neuroanatomical structures in SZ and schizoaffective disorder.

Using a multi-site dataset, Weiqi et al. [106] examined the brain age gap (BAG) as a possible indicator of SZ utilizing sMRI data from eight sites. They used support vector regression (SVR) to estimate age based on gray-matter volume and discovered that patients had much higher BAG than did controls, indicating its potential as a biomarker for SZ. Using a multisite dataset comprising COBRE [56], NMorphCH [60], and NUSDAST [58], Pierrefeu et al. [107] investigated the application of ML with structured sparsity to predict SZ in an interpretable and stable manner. They created a model that is both interpretable and replicable across sites.

Using the dataset from the Institute of Mental health (IMH), Singapore [108], Chilla et al. [109] used ensemble ML algorithms and a wide range of neuroanatomical markers to distinguish between SZ patients and HCs. The study’s classification accuracies ranged from 83 to 87%, with sensitivities and specificities of 90–98% and 65–70%, respectively. Using a multisite dataset from NUSDAST [58] and IMH [108], M. Hu et al. [110] used 3D convolutional neural networks (CNNs) to classify SZ and controls using brain MRI. The CNNs outperformed classic handcrafted feature-based ML algorithms in terms of accuracy, highlighting their promise for individual psychiatric diagnosis.

Table 4 provides a table summarizing the study characteristics of the key research on the sMRI prediction of SZ.

**Table 4:** Study characteristics of notable research relevant to SZ diagnosis utilizing sMRI (patients—patients with SZ).

Work	Dataset	Number of Samples	Data Preparation	Software for Data Preparation	Feature Extraction Method	Approach	Result (%)
Qureshi et al. [85]—2017	COBRE	Patients = 72, controls = 72	Data segmentation, group independent component analysis (GICA)	FreeSurfer, Analysis of Functional NeuroImages (AFNI), FMRIB Software Library (FSL) https://fsl.fmrib.ox.ac.uk (accessed on 10 September 2024).	Shape characteristics	ELM, nested 10-by-10-fold cross-validation	Accuracy = 99.29
Chen et al. [88]—2020		Patients = 34, controls = 34	Data segmentation	Statistical parametric mapping	GM and WM features	SVM, leave-one-out cross-validation (LOOCV)	Accuracy = 85
Tanveer et al. [90]—2022		Patients = 72, controls = 74	Smoothing, resampling	Computational Anatomy Toolbox (CAT12)	GM, WM and integrated GM & WM features, Wilcoxon Method	Neural network, 10-fold cross-validation	Accuracy = 86.71
Liu et al. [94]—2018	Clinical	Patients = 62, controls = 33	-	Functional magnetic resonance imaging of the brain (FMRIB)	Cortical GM volume, cortical thickness, fractional anisotropy	SVM, 6-fold cross-validation	Accuracy = 91.28
A V Nimkar et al. [95]—2018		-	Normalization	–	Features from multiple modalities, Boruta algorithm	SVM	Accuracy = 94.12
Nguyen et al. [96]—2018		Patients = 171, controls = 142	Data segmentation	Statistical parametric mapping	GANs	10-fold cross-validation	Accuracy = 99.3
Srinivasgopalan et al. [97]—2019		Patients = 69, controls = 75	ICA	-	deep CNN	Sigmoid	Accuracy = 94.44
Rokham et al. [99]—2020	B-SNIP	BPD = 176 patients = 374, controls = 362	Data segmentation	SPM	Brain image voxels, analysis of variance (ANOVA)	SVM	Accuracy = 89
Junhao et al. [101]—2022	SchizConnect	Patients = 450, controls = 437	skull striping, brain-and-head affine registration	Brain extraction tool	Hierarchical spatial features	3D-CNN	Accuracy = 92.1
Manohar et al. [103]—2018	NAMIC	Twenty groups	Data segmentation	-	Multi-objective BPSO	Fuzzy SVM	Accuracy = 90
Latha et al. [105]—2021	NAMIC and SchizConnect	Patients = 81, controls = 82	Data segmentation	-	BPSO	3-fold cross-validation	Accuracy = 90.09
Kadry et al. [111]—2021	Working Memory Dataset	99 subjects	-	-	Features selected by the slime module algorithm (SMA) VGG-16	SVM-cubic, 10-fold cross-validation	Accuracy = 94.33
Weiqi et al. [106]—2021	3 datasets from local institute, 5 datasets from SchizConnect	Patients = 501, controls = 512	Bias-field correction, segmentation, normalization, resampling, smoothing	SPM12, CAT12	Demographic features	SVR, 5-fold cross-validation	Accuracy = 95
Chilla et al. [109]—2022	Institute of Mental Health (IMH), Singapore	Patients = 158, controls = 76	Segmentation, cortical surface reconstruction, cortical parcellation	FreeSurfer	Cortical thickness, cortical surface area, and mean curvature	Ensemble of SVM, nuSVM, and LR 3-fold cross-validation	Accuracy = 87

### 4.3. fMRI-Based Diagnosis of SZ

**Figure 20:**
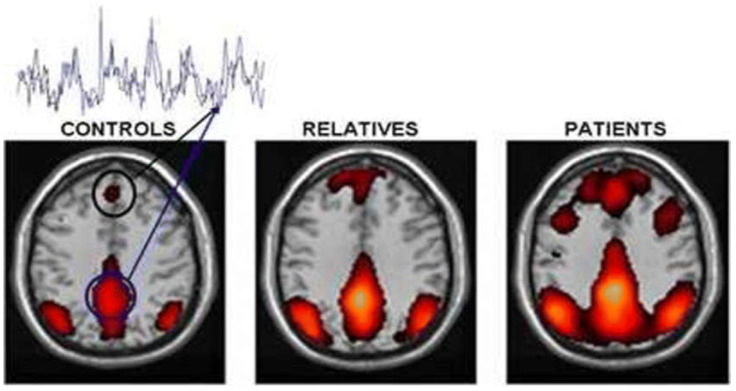
SZ diagnosis utilizing fMRI [29].

Early detection of brain disorders relies heavily on functional network changes. Studies have employed ML on fMRI scans to detect SZ caused by brain abnormalities [84]. In Figure 20, the default brain network is changed in SZ compared to HC [29].

**Figure 21:**
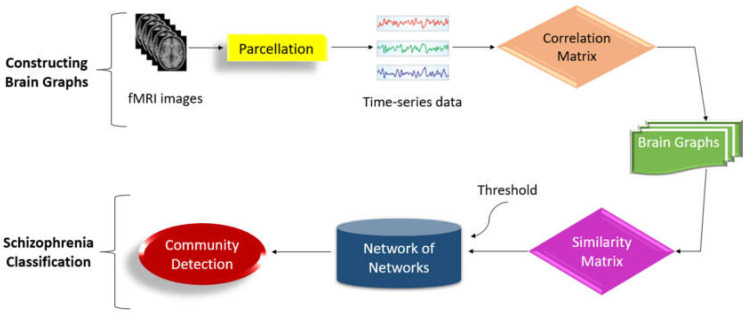
Architecture diagram of the study proposed by Nallusamy et al. [114].

Using the COBRE dataset [56], Qureshi et al. [13] proposed a methodology for distinguishing SZ patients from HCs using resting-state fMRI data. To extract and evaluate features from fMRI data, the authors used 3D-CNN in conjunction with ICA. The proposed technique had a ten-fold cross-validated classification accuracy of 98.09% and an AUC of 0.99821. Xiang et al. [112] proposed a method for detecting SZ utilizing graph with multiple view measurements regarding the functionality of brain structures. The authors presented an enhanced SZ identification method based on graphs with multiple view measurements of brain structures. Using the Brainnetome atlas, they created distinct functional connection networks for each patient. Multi-view graph measurements were then calculated using feature representations. Sparse group lasso was used to choose discriminative features by taking into account correlations between measures within the same brain area. A SVM classifier was utilized to identify SZ. The proposed method achieved an accuracy of 93.10% for SZ detection using the LOOCV scheme. Ghanbari et al. [113] created a DL system which integrates a 3D convolutional neural network (CNN) with a long short-term memory (LSTM). They pre-processed the fMRI images, identified functional connectivity elements, and used those to generate activity maps. These maps were then employed as inputs for the 3D CNN-LSTM model, which classified the images into healthy and schizophrenic groups. The model scored a 92.32% classification accuracy. Nallusamy et al. [114] developed a technique to distinguish between SZ patients and healthy participants using fMRI data. The fMRI scans underwent preprocessing to reduce noise. The automated anatomical labelling atlas was used to divide the human brain into 116 distinct areas. A region connection matrix was created using the fMRI data. The connectivity matrix yielded a weighted undirected graph. The graph similarity algorithm calculated the similarity between each graph or subject. A weighted network was established, with each graph representing a node and the edges made with the top k similarity scores. A community detection method was used to distinguish between SZ and normal people. Figure 21 shows the block diagram of the system. This approach comprises two phases: brain graph building from fMRI and SZ categorization.

Using the FBIRN dataset [64], Juneja et al. [115] focused on using fMRI data for computer-aided SZ diagnosis. The work employed two balanced datasets (D1 and D2) from the FBIRN multisite dataset [64], which include fMRI data from age-matched healthy volunteers and SZ patients undertaking an auditory oddball task. D1 and D2 were collected with 1.5 T as well as 3 T scanners, respectively. Voxels from three-dimensional spatial maps were segmented into anatomical brain regions. After the selection of a smaller collection of discriminative characteristics, the features were then fed into a SVM classifier, and performance was measured utilizing LOOCV. Their approach had a classification accuracy of 95.6% for D1 and 96.0% for D2. Chatterjee et al. [116] employed fMRI data collected during an auditory oddball task from both SZ patients and HCs. The authors proposed a three-stage feature selection model: the general linear model (GLM) was used to determine initial features, statistical hypothesis testing was used to improve the feature set, and a limited collection of approximately fifty discriminative features was selected, the selected features were then utilized to classify fMRI data from healthy controls and SZ patients. The proposed technique had a high classification accuracy of 99.5%.

Using the clinical dataset [76], Liu et al. [117] investigated aberrant brain functionality as a possible indicator of SZ utilizing ReHo and SVM analysis. This approach was used to assess neuronal activity synchronization in local brain areas. Support vector machine, an ML technology, was used to categorize patients and controls based on their brain activity patterns. The SVM model achieved 89.87% classification accuracy. Wang et al. [118] investigated the role of regional homogeneity (ReHo) as an imaging biomarker for adolescent-onset SZ. The study analyzed resting-state fMRI data from teenagers with SZ and HCs. To assess local brain activity synchronization, the scientists analyzed the fMRI data using ReHo. They used an SVM to classify SZ patients and HCs based on ReHo values. The study found that ReHo could efficiently distinguish between adolescent-onset SZ patients and HCs, indicating its potential as an imaging biomarker. Zhu et al. [119] proposed a method for detecting SZ using positive distinctive functionality of brain structures relevant to fMRI data. The authors presented a unique approach which identified changes in brain functional connectivity between SZ patients and HCs. For SZ classification, the suggested technique achieved 100% sensitivity, 90.48% specificity, and 95.56% overall accuracy. Salvador et al. [120] investigated MRI-based SZ diagnosis using multimodal brain image integration. The task-based fMRI activation levels, amplitude of low-frequency fluctuations, and weighted global brain connectivity were obtained from a single MRI session. They investigated multimodal techniques such as post-classification integration, two-step sequential integration, and voxel-level integration via one-dimensional CNNs (1D-CNNs). The lasso on the two-back task maps had a maximum unimodal classifier accuracy of 84%. Qiu et al. [121] investigated the classification of SZ patients and HCs by integrating ICA and CNNs. ICA obtained the relevant components of fMRI, which were later refined by CNNs to develop hierarchical diagnostic features and classify participants. The proposed method attained an average accuracy of 91.32% for subject-level classification using majority voting and 98.75% in the auditory cortex for slice-level classification. Niu et al. [122] investigated the use of sample augmentation to improve the classification of SZ patients and HCs from fMRI data. The authors integrated ICA and CNNs. They used sample augmentation techniques to improve the training process by creating new data samples from the existing dataset. The proposed method had an average classification accuracy of 91.32% for subject-level classification using majority voting.

Yang et al. [123] explored modern neuroimaging techniques to investigate functional connectivity patterns in the brain. They employed statistical and computational tools to discover connection deficits associated with SZ. The study found substantial results on the widespread nature of connectivity deficits in SZ. Using the CCNMD dataset [124], Y. Bae et al. [125] used ML to evaluate fMRI data and distinguish between SZ patients and HCs. The study used global and local network connectivity metrics and produced a high prediction accuracy of 92.1% with SVM. Lei et al. [126] utilized ML and multimodal neuroimaging for identifying SZ on an individual basis. The study achieved a classification accuracy of 90.83%.

Using the MCIC [61] and a genes dataset, Alam et al. [127] proposed a kernel machine method for detecting higher-order interactions in multimodal datasets, which was specifically applied to SZ. The authors created a semiparametric technique using a replicating kernel Hilbert space. This method was developed using a typical mixed-effects linear model. The strategy centered on recognizing interactions between many modalities (e.g., brain imaging and genetic data) in order to uncover complicated links with SZ. The suggested method outperformed established methods in finding higher-order interactions, indicating its potential for better understanding the multidimensional nature of SZ. Using the same dataset, Li et al. [128] offered a unique method for combining imaging and genomics data using deep principal correlated autoencoders. The study used imaging data (such as MRI) and genomics data from multiple sources to investigate the integration of these modalities. The authors proposed the deep principal correlated autoencoder (DPCAE) model. This model took advantage of principal component analysis (PCA) and autoencoders to capture the associated characteristics of imaging and genomics data. The DPCAE model was intended to improve the representation and integration of multimodal data. The proposed technique showed better performance in merging imaging and genomics data.

Table 5 provides a table summarizing the study characteristics of key research on the use of fMRI to predict SZ.

**Table 5:** Study characteristics of notable research relevant to SZ diagnosis using fMRI (patients—patients with SZ).

Work	Dataset	Number of Samples	Data Preparation	Software for Data Preparation	Feature Extraction Method	Approach	Result (%)
Qureshi, et al. [13]—2019	COBRE	Patients = 72, controls = 72	Independent component analysis	FMRIB Software Library	Three-dimensional–convolutional neural network	10-fold cross-validation	Accuracy = 98.09, recall = 97.49
Ghanbari et al. [113]—2023		Patients = 72, controls = 74	Motion correction, spatial normalization, smoothing	SPM12	Functional connectivity features, activity maps, statistical methods	3D-CNN, LSTM network	Accuracy = 92.32
P. Jain et al. [129]—2021	UCLA COBRE	Patients = 71, controls = 73	Slice-time correction, realignment, co-registration, normalization, smoothing	SPM12	FC map features, two-sampled paired *t*-test	2- step ridge Classifier, LOOCV	Accuracy = 85.26
A. Juneja et al. [115]—2018	FBIRN	Patients = 34, controls = 34 Patients = 25, controls = 25	GSICA and GLM	GIFT and SVM	Filter-cum-wrapper method	SVM	Accuracy = 96
I. Chatterjee et al. [116]—2018		Patients = 55, controls = 55	-	SPM	3-stage algorithm	Linear support vector machine	Accuracy = 99.5
Chatterjee et al. [130]—2020		Patients = 55, controls = 55	-	SPM	Voxel values T-rest, GLM, NSGA-II	SVM	Accuracy = 95.45
S. Wang et al. [118]—2018	Clinical	Patients = 48, controls = 31	-	REST	Regional homogeneity measures	Support vector machines	Accuracy = 90.14
Q. Zhu et al. [119]—2018		Patients = 24, controls = 21	-	Statistical parametric mapping	Features of brain connectivity, KDA	Neural Networks	Accuracy = 95.56
P. Liu et al. [131]—2019		Patients = 28, controls = 28	Group ICA	Representational state transfer (REST)	Nodes of FCN, PCA	SVM, LOOCV	Accuracy = 92.86
Yang et al. [123]—2017	Multiple sites	Patients = 446, controls = 451	-	Statistical parametric mapping	Sparsity parameter	10-fold cross-validation	Accuracy = 86
Y. Bae et al. [125]—2018	CCNMD	Patients = 21, controls = 54	-	Statistical parametric mapping	Parameters of functional connectivity	10-fold cross-validation	Accuracy = 92.1, precision = 94, recall = 92
Lei et al. [126]—2020	Five datasets	Patients = 295, controls = 452	Data segmentation	DPARSF	Volume of gray and white matter, covariance matrix, fuzzy C-means (FCM) clustering, ReHo	SVM, 10-fold cross-validation	Accuracy = 90.83
Alam et al. [127]—2018	Genes dataset of MCIC	Patients = 79, controls = 103 75 genes	-	Statistical parametric mapping	epigenetics features, kernel-Based multi-view discriminative and high-order information (KMDHOI)	SVM, 2-fold cross-validation	Accuracy = 89.68
G. Li et al. [128]—2020	MCIC	Patients = 81, controls = 103	Data augmentation and data segmentation	Statistical parametric mapping	DPCAE	5-fold correlated AE	Accuracy = 93.8

## 5. Critical Analysis and Discussion

This section presents a critical review of the papers surveyed in the report. After a detailed debate, we identified significant difficulties that require further attention from researchers for the better diagnosis of SZ. Some of these concerns are outlined below:

1. EEG signals are adulterated by various inputs, such as eye blinks, muscle movements, and electrical noise, which can obscure the true neural signals, hindering the ability to examine the data accurately. For ensuring the reliability of EEG investigations, it is critical to develop standardized and effective methods for artifact removal. Techniques such as ICA and ML-based approaches are currently being investigated, although a consensus on best practices is still required.
2. EEG is noted for its excellent temporal resolution, which can capture cerebral activity in milliseconds. However, its spatial resolution is limited, creating challenges in identifying the specific site of brain function. Combining EEG with other imaging modalities, such as fMRI, which has excellent spatial resolution, can provide a more complete picture of brain activity. However, combining various modalities necessitates complicated algorithms and approaches that are still being developed.
3. EEG data preprocessing steps include filtering, artifact removal, and normalization. There is no commonly approved preprocessing pathway, resulting in diversity in study outcomes. Developing uniform preprocessing techniques would improve the reliability and comparability of EEG research.
4. The ability of DL algorithms to evaluate EEG data for SZ detection has been demonstrated. However, these models require substantial training datasets, which are not always available. Furthermore, DL models are sometimes viewed as “black boxes,” creating challenges in understanding the predictions. Creating interpretable models and tackling data shortage issues are critical concerns.
5. SZ is linked to modest structural abnormalities in the brain, such as variations in gray-matter volume and cortical thickness. These modifications might vary greatly between individuals, making it difficult to create models that accurately capture these differences. More complex models can be built using advanced ML techniques and larger datasets.
6. Most sMRI investigations are cross-sectional, capturing brain structure at a single point in time. Longitudinal studies that track structural changes over time are required to better understand the course of SZ. These investigations can shed light on how brain structure changes over time and aid in the identification of early illness biomarkers.
7. Combining sMRI with functional data from fMRI or EEG can help provide a more complete picture of SZ. However, effective integration strategies are still being developed. Multimodal techniques can assist in establishing the link between anatomical abnormalities and functional problems in SZ.
8. fMRI has high spatial resolution, allowing researchers to identify the location of brain activity. On the other hand, its temporal precision being restricted, it measures fluctuations in the activity of the brain over seconds instead of milliseconds. Improving the temporal resolution of fMRI and better combining it with EEG can provide a more thorough picture of brain function.
9. There is ongoing disagreement concerning whether task-based or resting-state fMRI is more effective in diagnosing SZ. Task-based fMRI requires participants to execute specific tasks, whereas resting-state fMRI monitors brain activity at rest. Each methodology has merits and demerits; hence, additional studies are required in order to establish the most efficient method for detecting SZ.
10. fMRI data are complicated and multidimensional, necessitating modern computer approaches for analysis. Meaningful patterns can be extracted from fMRI data using methods such as ML and network analysis. However, more robust and interpretable models are needed to provide unambiguous insights into brain activity in SZ.
11. Combining data from EEG, sMRI, and fMRI can provide a more complete picture of brain function and anatomy. However, successful multimodal integration techniques are still in their infancy. Developing algorithms that can seamlessly combine data from several modalities is an important field of research.
12. There is a dearth of consistent processes for data collection, preparation, and analysis among investigations. This diversity makes it difficult to compare data and draw broad conclusions. Standardized methods would improve the repeatability and comparability of study results.
13. Many advanced models, particularly those based on DL, are difficult to comprehend. Developing models that provide precise insights into the underlying brain pathways is critical for comprehending SZ. Interpretable models can aid in the identification of biomarkers and a better knowledge of disease pathology.
14. Large, high-quality datasets are required to train robust models. More open-access datasets are required, as are collaborative data-sharing efforts. Initiatives such as data-sharing platforms and consortia can help to overcome data scarcity and facilitate the creation of more accurate models.
15. AI algorithms, particularly deep learning models, often face overfitting issues when applied to small datasets, which is common in schizophrenia research due to challenges in acquiring large-scale EEG or neuroimaging data. Techniques such as data augmentation, transfer learning, and regularization can mitigate this problem, but their effectiveness depends on the specific dataset and application.
16. Despite promising advancements, deploying AI systems in clinical environments presents several practical challenges. These include integrating AI solutions into existing workflows, ensuring data privacy and security, and meeting stringent regulatory requirements. Rigorous validation, pilot testing in clinical settings, and collaboration with healthcare professionals are critical to overcoming these obstacles and ensuring the reliability of AI systems for schizophrenia detection.

These points highlight some of the important areas where additional study and development is needed to better the diagnosis and understanding of SZ using EEG, sMRI, and fMRI. Table 6 highlights the highest classification accuracies achieved by the corresponding datasets notable for SZ diagnosis.

**Table 6:** Highest classification accuracies attained on the respective datasets that are relevant for SZ diagnosis.

Work	Dataset	Modality	AI Technique	Highest Classification Accuracy (%)
Buettner et al. [72]—2019	RepOD	EEG	RF, 10-fold cross-validation	100
Siuly et al. [70]—2023	Kaggle Basic Sensory Task data	EEG	Deep ResNets, Softmax layer and deep features with SVM	99.23
Sobahi et al. [83]—2022	MSU	EEG	ELM-based AE	97.7
Qureshi et al. [85]—2017	COBRE	sMRI, fMRI	ELM	99.29
Nguyen et al. [96]—2018	Clinical	sMRI	SVM, 10-fold cross-validation	99.3
Chatterjee et al. [116]—2018	FBIRN	fMRI	Linear SVM	99.5

## 6. Conclusions and Future Scope

AI developments have opened up new potential for detecting mental diseases such as SZ. SZ, being a brain dysfunction, involves hallucinations, aberrant speech, and cognitive, thinking, and behavioral impairments. This study examined AI methods such as feature reduction, selection, ML, and DL for diagnosing SZ automatically. We reviewed previous studies on utilizing ML and DL to diagnose SZ and promote healthy behavior. Recent studies have demonstrated high accuracy in prediction tests, sparking interest in AI techniques.

The paper discusses three key modalities used to detect SZ: EEG, sMRI, and fMRI. Recent studies have emphasized the use of MRI data for SZ categorization above EEG data due to its ability in providing critical analysis of the functionality of the brain and promoting accurate diagnosis. While sMRI is commonly used to classify patients into SZ and HC groups, resting-state fMRI is increasingly being used by researchers.

Researchers can use public datasets to improve diagnoses with ML and DL models. Medical datasets and AI approaches can enhance the prognosis for chronic illnesses such as SZ. DL demands massive quantities of data for complex tasks. However, due to limited datasets, ML is more commonly used for automated SZ diagnosis. While ML can be utilized with limited datasets, DL algorithms with autonomous feature selection can help doctors understand disease causes more effectively. DL can predict initial onset of SZ as well as identify a significant treatment option. Our examined research stresses the importance of using a balanced and context-specific strategy. AI has the potential to improve SZ diagnosis, but it requires interdisciplinary collaboration and ethical considerations for its implementation in mental health.

Future research could benefit from incorporating generative models to enhance schizophrenia detection and classification. Techniques such as generative adversarial networks (GANs) and variational autoencoders (VAEs) may be used to generate synthetic EEG or neuroimaging data, helping to overcome the challenge of limited datasets in psychiatric studies. By supplementing real data with high-quality synthetic samples, these models can strengthen the performance of classification algorithms and improve their ability to generalize. Furthermore, they can identify intricate patterns in brain activity, offering valuable insights into the neural mechanisms underlying schizophrenia. Investigating the use of generative models has the potential to not only boost diagnostic accuracy but also introduce innovative analytical methods and simulations, advancing our understanding of this complex disorder.

## Data Availability

No new data were created or analyzed in this study.

## Supplementary Materials

The following supporting information can be downloaded at: https://www.mdpi.com/article/10.3390/diagnostics14232698/s1, Table S1: PRISMA 2020 Checklist. Reference [54] is cited in Supplementary Materials.
